# A Split-Gate Positive Feedback Device With an Integrate-and-Fire Capability for a High-Density Low-Power Neuron Circuit

**DOI:** 10.3389/fnins.2018.00704

**Published:** 2018-10-09

**Authors:** Kyu-Bong Choi, Sung Yun Woo, Won-Mook Kang, Soochang Lee, Chul-Heung Kim, Jong-Ho Bae, Suhwan Lim, Jong-Ho Lee

**Affiliations:** Department of Electrical and Computer Engineering, Inter-University Semiconductor Research Center, Seoul National University, Seoul, South Korea

**Keywords:** neuromorphic, positive feedback, steep subthreshold swing (*SS*), integrate-and-fire (I&F), spiking neural network (SNN), unsupervised learning, pattern recognition

## Abstract

Hardware-based spiking neural networks (SNNs) to mimic biological neurons have been reported. However, conventional neuron circuits in SNNs have a large area and high power consumption. In this work, a split-gate floating-body positive feedback (PF) device with a charge trapping capability is proposed as a new neuron device that imitates the integrate-and-fire function. Because of the PF characteristic, the subthreshold swing (*SS*) of the device is less than 0.04 mV/dec. The super-steep *SS* of the device leads to a low energy consumption of ∼0.25 pJ/spike for a neuron circuit (PF neuron) with the PF device, which is ∼100 times smaller than that of a conventional neuron circuit. The charge storage properties of the device mimic the integrate function of biological neurons without a large membrane capacitor, reducing the PF neuron area by about 17 times compared to that of a conventional neuron. We demonstrate the successful operation of a dense multiple PF neuron system with reset and lateral inhibition using a common self-controller in a neuron layer through simulation. With the multiple PF neuron system and the synapse array, on-line unsupervised pattern learning and recognition are successfully performed to demonstrate the feasibility of our PF device in a neural network.

## Introduction

Conventional computing systems based on von-Neumann architecture suffer from an energy efficiency problem compared to biological brains in processing the complex data and information (Cantley et al., 2011; Indiveri et al., 2011; Yu et al., 2011; Park et al., 2013). As an alternative to conventional computing architectures, neuromorphic computing architectures have been studied to enable complex processes, such as pattern recognition, classification, and perception (Wijekoon and Dudek, 2008; Ghosh-Dastidar and Adeli, 2009; Basu et al., 2013; Rajendran et al., 2013; Kasabov, 2014; Eryilmaz et al., 2015). Among these architectures, deep neural networks (DNNs) such as deep-belief network (DBN) and convolutional network (ConvNet), which are computing architectures that use a mathematical algorithm model, have been widely reported to reduce the computing energy by mimicking the parallel computation of biological brains (Hinton et al., 2006; Turaga et al., 2010; LeCun et al., 2015; Krizhevsky et al., 2017; Liu et al., 2017). However, software-based DNNs still have energy efficiency issues due to the energy consumed by the synchronous information processing and data movement between the processor and memory such as static random access memory (SRAM) and dynamic random access memory (DRAM) (Chen et al., 2017). On the other hand, spiking neural networks (SNNs) based on event-driven sparse communication and local learning without using external memory can reduce the power consumption, and many studies have been reported (Kim et al., 2015; Moon et al., 2015; Qiao et al., 2015; Park and Lee, 2017). The SNNs mimic the human brain more closely, consisting of synapses and neurons, and provide a highly compact model for building a larger-scale neuromorphic system platform. The function of the “integrate-and-fire (I&F) neuron model,” which is the fundamental part of the SNNs, is that a neuron fires when integrated signals exceed a specific threshold. To implement a hardware-based I&F function in SNNs, various types of neuron circuits such as a log-domain LPF neuron, differential pair integrator and fully digital I&F circuit have been widely reported (Indiveri et al., 2011). However, complementary metal-oxide semiconductor (CMOS) based neuron circuits for complex neuron functions require many transistors and large capacitors including a membrane capacitor (*C*_mem_) in a neuron circuit leading to a large area. Therefore, to implement a high density SNN, neuron devices with the integration capability have recently been reported, such as impact-ionization based NPN selector (I-NPN) and NPN device with an extended gate (gated-INPN) on a silicon-on-insulator (SOI) platform (Ostwal et al., 2015; Dutta et al., 2017), spin-transfer torque (STT) devices based on a magnetic tunnel junction (MTJ) (Sharad et al., 2012; Zhang et al., 2016) and memristor (Wang et al., 2018). However, the neuron circuit based on the SOI devices using the impact-ionization has the body-floated CMOS FETs, and their performance can be degraded by the floating-body effect (Vandana, 2013). The stochastic switching characteristic of the STT device can cause unwanted fires in neurons with integrated signals that do not exceed the threshold, and most of the materials that make up the device are not compatible with the CMOS process. The memristor is compatible with the CMOS process, but it has reliability issues when manufactured in a nanoscale and constructed as multiple array layers (Pouyan et al., 2014).

In addition, to achieve a low power neuron circuit, a device with steep switching characteristics is required. The switching characteristic of conventional MOSFETs is limited by the theoretical limit of the subthreshold swing (*SS*) of 60 mV/dec at room temperature (*T* = 300 K) (Cheung, 2010). To overcome the switching limitation of conventional MOSFETs, many steep *SS* devices have been reported, such as an impact-ionization MOS (I-MOS) using the avalanche break down (Choi et al., 2005), band-to-band tunneling FET (TFET) (Choi et al., 2007) and PNPN based-feedback FETs (FB-FETs) (Wan et al., 2013; Jeon et al., 2015; Dirani et al., 2017). These steep *SS* devices are fabricated using SOI wafers because they require a floating-body to reduce leakage or to accumulate carriers. However, the performance of body-floated CMOS FETs fabricated on a SOI wafer with steep *SS* device can be degraded by the floating-body effect and self-heating effect (Su et al., 1994).

In this paper, we propose, for the first time, a split-gate floating-body positive feedback (PF) device based on a gated PNPN structure for the I&F function and introduce key fabrication steps for the PF device fabricated using the CMOS process. For an I&F neuron circuit, body-connected CMOS FETs are fabricated on the same wafer with a body-floated PF device. The fabricated devices are investigated in terms of the fundamental device properties and neuron operation. With the PF device, we also demonstrate the I&F, reset and lateral inhibition functions of the SNN system using a circuit simulator and performed on-line unsupervised pattern learning and recognition using a MATLAB simulator.

## Materials and Methods

### Device Fabrication and Integration

**FIGURE 1:**
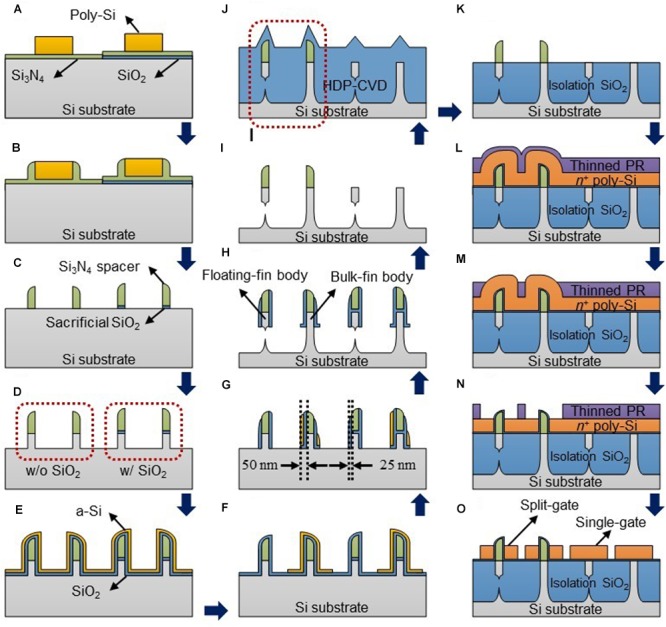
**(A–O)** Schematic cross-sectional views of key process steps of the fabricated positive feedback (PF) device or the split-gate floating-body FET (leftmost in each figure), the split-gate bulk FET, the single-gate floating-body FET and the single-gate bulk FET (sequentially from the left in each figure) on the same substrate.

The PF device and several CMOS FETs were fabricated on the same (100) bulk Si wafer using 11 masks and unit processes of the CMOS technology. **Figure 1** shows schematic cross-sectional views of key process steps for the PF device, the split-gate floating-body FET, and the split-gate bulk FET, the single-gate floating-body FET and the single-gate bulk FET. A thin sacrificial silicon dioxide (SiO_2_) was deposited and patterned. A thin silicon nitride (Si_3_N_4_) was deposited to passivate the sacrificial SiO_2_. Then, a thick poly-Si layer was deposited and patterned (**Figure 1A**), followed by Si_3_N_4_ deposition and etching for the spacer formation (**Figure 1B**). The poly-Si was stripped, and the SiO_2_/Si_3_N_4_ not protected by the Si_3_N_4_ spacer was etched (**Figure 1C**). Through these process steps, the Si_3_N_4_ spacers with and without the sacrificial SiO_2_ were formed for the split-gates and single-gate formation on the same wafer. Si fins were formed by dry etching using the Si_3_N_4_ spacer as a hard-mask (**Figure 1D**). After forming the SiO_2_/amorphous silicon (a-Si) (25 nm/25 nm) stack (**Figure 1E**), only the a-Si was patterned to form sidewall masks with different thicknesses using a photoresist (PR) mask (**Figure 1F**). Then, the SiO_2_ and the SiO_2_/a-Si stack were anisotropically etched followed by isotropic etching of the exposed Si (**Figures 1G,H**). Note that only the Si fins with thinner sidewall masks were floated by controlling the etching time with SF6 gas leading to the formation of the floating fin and bulk fin bodies on the same substrate. By wet etching all of the SiO_2_ in a buffered hydrogen fluoride (BHF) solution, the SiO_2_ sidewall masks and only the Si_3_N_4_ spacers formed on the sacrificial SiO_2_ were removed (**Figure 1I**). A thick SiO_2_ layer was deposited by a high-density plasma chemical vapor deposition (HDP-CVD) process and wet etched partly to form an isolation oxide (**Figures 1J,K**). A thin SiO_2_ buffer layer was deposited to protect against ion implant damage followed by ion implantations for the control of the threshold voltage under the isolation oxide, electron injection barrier (p-type doping, Np), and n-/p-well doping. After removing the buffer SiO_2_, a tunneling SiO_2_/Si_3_N_4_/blocking SiO_2_ (O/N/O) gate insulator stack was formed for the charge trap layer, and a heavily doped n-type (n+) polysilicon (poly-Si) was deposited for the gate formation. By coating the wafer with a thinned PR (2:1 mixture of PR and thinner), the PR on the n+ poly-Si deposited on the Si_3_N_4_ spacers was much thinner than that on the n+ poly-Si without the Si_3_N_4_ spacers (**Figure 1L**). After partly etching the thinly coated PR, only the n+ poly-Si on the Si_3_N_4_ spacers was exposed (**Figure 1M**). Then, the exposed n+ poly-Si was etched partly to split the n+ poly-Si on both sides of the Si_3_N_4_ spacer (**Figure 1N**). Then, the thinned PR was removed followed by the n+ poly-Si patterning to form the gates (**Figure 1O**). The split-gates and single-gates were formed on the same wafer. The O/N/O stack and the Si_3_N_4_ spacer in the ungated region were removed. After a buffer SiO_2_ was grown on the Si fins, ion implantations were performed for the source/drain (S/D) or anode/cathode and hole injection barrier doping (n-type doping, Nn). This was followed by a rapid thermal annealing (RTA) at a temperature of 1050°C for 5 s. Then, an inter-layer dielectric (ILD) was deposited, and contact holes were patterned. A nickel (Ni)/titanium nitride (TiN) stack was deposited, and silicidation was performed by annealing at a temperature of 450°C for 1 min for NiSi formation (Wu et al., 2007). After unreacted Ni and TiN were stripped in piranha solution (H2SO4/H2O2), TiN was deposited and patterned for the electrode followed by H2 annealing at 350°C for 10 min.

### Electrical Characterization

Electrical characterization of the fabricated PF device and the four different kinds of devices was carried out using a probe station (Cascade, Cascade Microtech), a semiconductor device analyzer (B1500A, Agilent Technologies) and a waveform generator and fast measurement unit (WGFMU) module. Simulated I–V curves and energy band diagrams were obtained from a TCAD simulator (Sentaurus, Synopsys).

### I&F Circuit and Multiple Neuron System

The I&F circuit with the PF device was simulated using a TCAD mixed-mode simulator (Sentaurus, Synopsys). In the mixed-mode simulations, the PF device was simulated by a device simulator, and the several CMOS FETs were simulated by a circuit simulator. The multiple neuron system including the PF neuron circuits, which have several CMOS FETs and an equivalent circuit reflecting the electrical behavior of the fabricated PF device, was simulated using a circuit simulator (HSPICE, Synopsys) with Predictive Technology Models (PTMs).

### Pattern Classification

The pattern classification was evaluated through simulations using the MATLAB software. The fully connected 2-layer SNN system was used to perform system-level verification of the pattern classification. The system consists of an input layer of 784 presynaptic neurons and an output layer of 10 postsynaptic neurons. The pattern classification was carried out in two steps: learning and recognition. Before the learning process, the weights of all the synapses were initialized to a random distribution. In the learning process, the synchronized binary 28 × 28 input pulses were applied to the synapse array. The behavioral modeling of synaptic devices and the proposed spike-timing-dependent plasticity (STDP) learning rule (Kim et al., 2018) were used for learning the synaptic weights. The I&F, reset and lateral inhibition functions were used for systematic operation of multiple neurons. In the recognition process, the I&F and reset functions were only used, and the firing rates of 10 output neurons were compared to identify which number is recognized.

## Results and Discussion

### Key Feature of the PF Device

**FIGURE 2:**
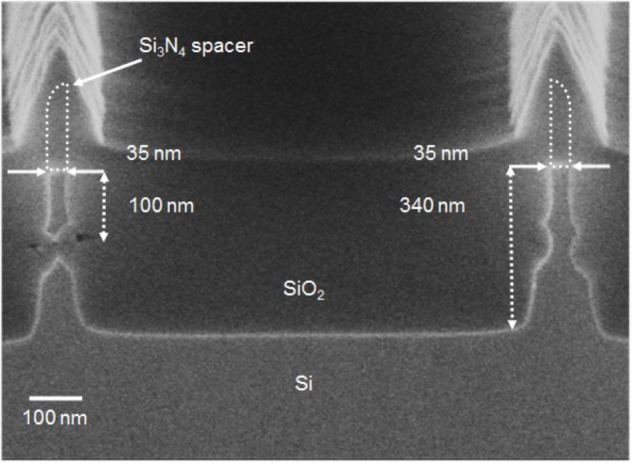
Cross-sectional SEM image of the floating-fin and bulk-fin bodies fabricated on the same substrate corresponding to the two structures in a dotted box shown in **Figure 1J**.

The PF device with steep switching characteristics using PF requires a floating-body channel to accumulate the injected electrons and holes to lower the hole and electron injection barriers, respectively. However, when neuron circuits including PF devices are integrated on the same substrate with CMOS FETs, these FETs have a floating-body, which may degrade the performance of the device. Therefore, a new process design should be made to enable the floating-body and the body connected to the substrate to be implemented on the same wafer. We successfully fabricated body-floated (floating-fin body) PF devices and body-connected (bulk-fin body) CMOS FETs on the same wafer (see section “Materials and Methods”). **Figure 2** shows a cross-sectional scanning electron microscopy (SEM) image of the floating-fin and bulk-fin bodies fabricated on the same substrate corresponding to the two structures in the dotted box shown in **Figure 1J**. *I*–*V* characteristics of the bulk-fin body CMOS FETs and several devices fabricated on the same wafer are shown in **Supplementary Figure S1**.

**FIGURE 3:**
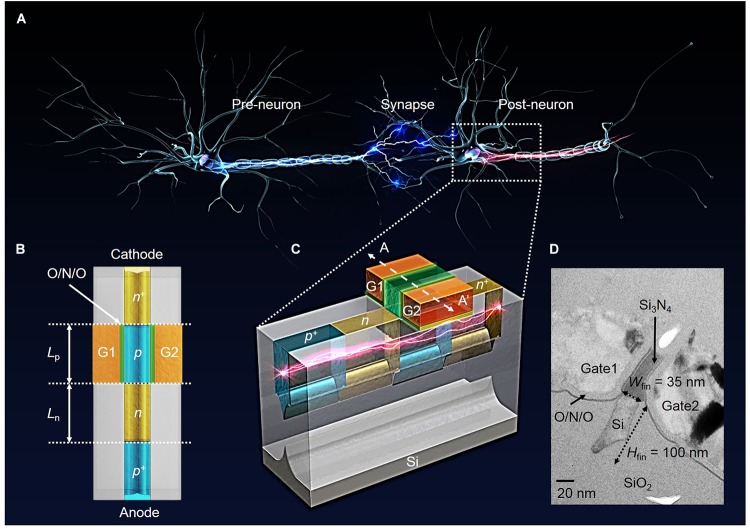
Schematic and cross-sectional views of the fabricated PF devices. **(A)** Three-dimensional illustration of biological neurons. **(B)** Top view of the PF device. **(C)** Three-dimensional schematic view of the fabricated PF device as a neuron device to mimic the I&F function of biological neurons. **(D)** Cross-sectional TEM image of the fabricated PF device cut along the dashed line A–A′ in **(C)**. Here, the width and height of the floating-fin body are 35 and 100 nm, respectively.

**FIGURE 4:**
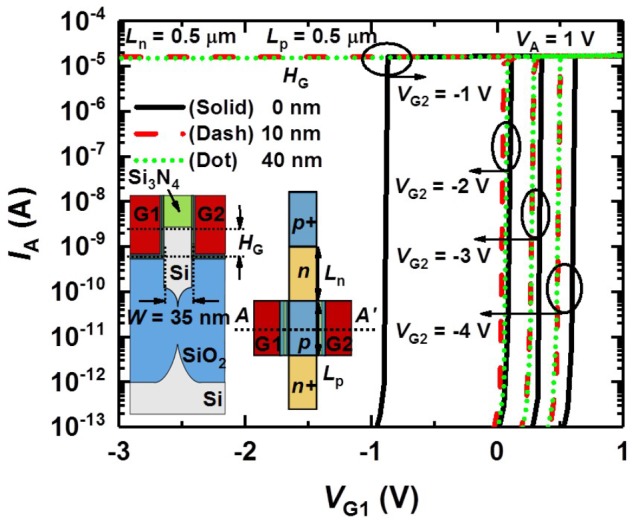
Simulated *I*_A_–*V*_G1_ curves of the PF device as parameters of the *H*_G_ and *V*_G2_ at a fixed *V*_A_ of 1 V. Here, the length of both the hole and electron injection barriers is 0.5 μm.

**Figures 3B,C** show the top and three-dimensional schematic views of the proposed PF device corresponding to part of the biological neuron depicted by the dotted box in **Figure 3A**. The PF device based on a gated PNPN structure has an anode, ungated region (*n*-type, hole injection barrier), gated region (*p*-type, electron injection barrier) and cathode region sequentially from the left shown in **Figure 3C**. Both the anode (*p*^+^) and cathode (*n*^+^) doping concentrations (or source/drain of the *p*-MOSFET and *n*-MOSFET, respectively) of the fabricated PF device are 2 × 10^20^ cm^-3^ for the ohmic contact between the TiN and the doped regions. The doping concentration of both hole and electron injection barriers is ∼1 × 10^18^ cm^-3^. **Figure 3D** shows a cross-sectional transmission electron microscopy (TEM) image of the fabricated PF device cut along the dashed line A–A′ in **Figure 3C**. The PF device has a split-gate floating-body structure. The *n*^+^ poly-Si gate1 (G1) and gate2 (G2) are located on both sides of the Si_3_N_4_ spacer. The floating-fin body width (*W*_fin_) and height (*H*_fin_) are 35 nm and 100 nm, respectively. The thickness of the O/N/O stack between the channel and gate for the charge trap layer is 2/4.2/9 nm. Here, the overlap height (*H*_G_) between the gate and Si fin is very small. The turn-on voltage (*V*_on_) of the PF device at which the anode current (*I*_A_) suddenly increases is weakly dependent on the *H*_G_ shown in **Figure 4**. Note that the *H*_G_ very slightly affects the *V*_on_ at a G2 bias (*V*_G2_) lower than -1 V. Thus, we adopted a *H*_G_ of 0 nm (nearly planar channel) to demonstrate the key concept of our PF device.

### I–V Characteristics of the Proposed PF Device

**FIGURE 5:**
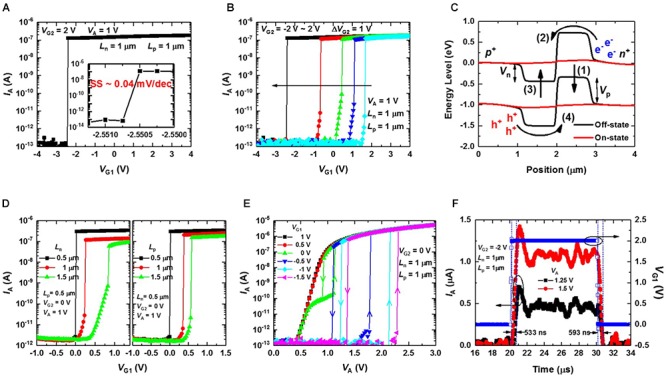
*I*–*V* characteristics of the proposed PF device. **(A)** *I*_A_–*V*_G1_ curve of the fabricated PF device at a *V*_G2_ of 2 V and a *V*_A_ of 1 V. The inset shows that the subthreshold swing (*SS*) of the device is less than 0.04 mV/dec. **(B)** *I*_A_–*V*_G1_ curves of the fabricated PF device as a parameter of *V*_G2_ (from –1 to 2 V) at a fixed *V*_A_ of 1 V. **(C)** Simulated energy band diagram of the PF device along the channel direction in the turn-off state (*V*_G1_ of –1 V) and turn-on state (*V*_G1_ of 3 V) at a *V*_G2_ of 0 V and a *V*_A_ of 1 V. **(D)** *I*_A_–*V*_G1_ curves of the fabricated PF device as parameters of the hole injection barrier length (*L*_n_) (left) and electron injection barrier length (*L*_p_) (right), respectively, at a *V*_G2_ of 0 V and a *V*_A_ of 1 V. As *L*_n_ and *L*_P_ increase, *V*_on_ increases. **(E)** *I*_A_–*V*_A_ curves of the fabricated PF device as a parameter of *V*_G1_ (from 1 to –1.5 V) at a fixed *V*_G2_ of 0 V. When *V*_G1_ < 0 V, *I*_A_ increases very rapidly when the *V*_A_ reaches the turn-on condition. **(F)** Transient *I*_A_ behaviors of the fabricated PF device when a single pulse is applied to gate1 (open symbol) while *V*_A_ is fixed at 1.25 and 1.5 V. All measurements were performed on the same device except for the devices in **(D)**.

**Figure 5A** shows the *I*_A_ versus G1 bias (*V*_G1_) curve of the fabricated PF device at a *V*_G2_ of 2 V and an anode bias (*V*_A_) of 1 V. The length of both the electron (*L*_p_) and hole (*L*_n_) injection barriers is 1 μm. The *SS* of the PF device is less than 0.04 mV/dec (resolution limit of the measurement system) shown in the inset of **Figure 5A**. As far as we know, the proposed PF device has the smallest *SS* compared to the other steep *SS* devices reported (**Table 1**). Additionally, *V*_on_ of the PF device can be modulated by the *V*_G2_ (**Figure 5B**). The abrupt increase of the current is due to the PF triggered by the applied *V*_G1_. The mechanism of the steep switching operation can be explained by an energy band diagram. **Figure 5C** shows the simulated energy band-diagram of the PF device along the channel in the turn-off state (*V*_G1_ of -1 V) and turn-on state (*V*_G1_ of 3 V) at a *V*_G2_ of 0 V and a *V*_A_ of 1 V. As the *V*_G1_ is increased, the electron injection barrier (*V*_p_) decreases (1), and electrons are injected into the hole injection barrier region (2) which leads to a decrease of the hole injection barrier (*V*_n_) (3). Then, the holes are injected into the electron injection barrier region (4) further reducing the *V*_p_ (1), and electrons are injected into the hole injection barrier region again. As this process repeats with PF, the device switches rapidly from the turn-off state to the turn-on state. However, as the *L*_n_ or *L*_p_ increases, the recombination of minority carriers increases leading to an increase of *V*_on_, a decrease of *I*_on_, and the degradation of the sharp switching characteristic (**Figure 5D**).

**Table 1:** Comparison of the electrical characteristics of steep *SS* devices.

Reference	Substrate	Operating Principle	|*V*_DS_| (V)	*I*_on_/*I*_off_	*SS* (mV/dec)
Choi et al., 2005	SOI	Avalanche breakdown	6.5	∼10^6^	3.7
Abele et al., 2005	Bulk	Mechanical switching	8.9	>10^3^	2.16
Choi et al., 2007	SOI	Band-to-band tunneling	1	∼10^4^	52.8
Jo et al., 2015	Bulk	Negative capacitance	2	∼10^5^	18
Wan et al., 2012, 2013	SOI	Positive feedback	1.5	∼10^8^	∼1
Jeon et al., 2015	Flexible	Positive feedback	1	∼10^6^	10
Dirani et al., 2016	SOI	Positive feedback	1-1.5	∼10^10^	8
Dirani et al., 2017	SOI	Positive feedback	1.2	∼10^8^	7
This work	Bulk	Positive feedback	1	∼10^6^	∼0.04

**Figure 5E** shows the *I*_A_–*V*_A_ curves as a parameter of *V*_G1_ at a fixed *V*_G2_ of 0 V. When *V*_G1_ > 0 V, *I*_A_ shows *p*-*n* diode properties because *V*_p_ is effectively decreased by the positively biased *V*_G1_. When *V*_G1_ < 0 V, *I*_A_ shows rapid switching due to the PF being triggered by the carriers generated in the reversed biased *p*-*n* junction as *V*_A_ increases. The generated holes and electrons accumulate in the electron and hole injection barrier regions, respectively, which leads to the decrease of *V*_p_ and *V*_n_. Then, electrons and holes are injected into the hole and electron injection barrier regions, respectively, to further reduce the *V*_n_ and *V*_p_. As this process repeats, the *I*_A_ shows sharp switching characteristics in the forward *V*_A_ scan. Hysteresis occurs when the reverse *V*_A_ scan time is shorter than the lifetime of the injected minority carriers after abrupt switching occurs in the forward *V*_A_ scan. The transient *I*_A_ behaviors when *V*_G1_ is switched from 0 to 2 V and again to 0 V at a fixed *V*_G2_ of -2 V are shown in **Figure 5F**. We can clearly see the turn-on and turn-off characteristics in the figure.

**FIGURE 6:**
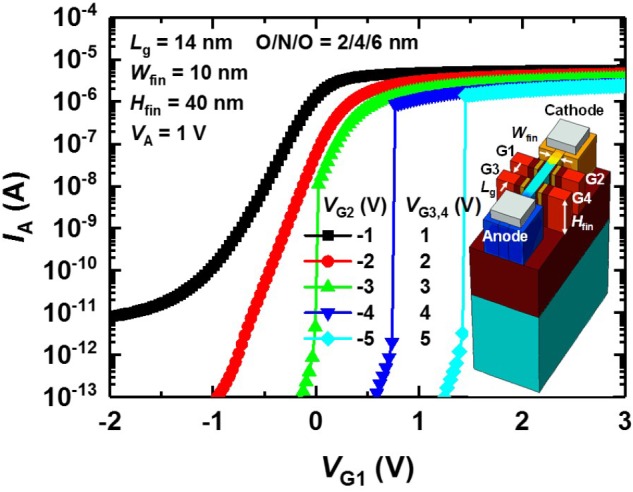
Simulated *I*_A_–*V*_G1_ curves of the scaled PF device. Here, *W*_fin_ = 10 nm and *H*_fin_ = 40 nm. The length (*L*_G_) of the gates (G1 ∼ G4) is 14 nm. The *p*-type doping concentration in the floating-fin body except for the anode and cathode is 2 × 10^15^ cm^-3^. The negative *V*_G2_ forms the electron injection barrier, and the positive *V*_G3_ and *V*_G4_ form the hole injection barrier.

We also demonstrate that the proposed PF device works at the 14 nm node by showing simulated *I*_A_–*V*_G1_ curves (**Figure 6**). Here, the *W*_fin_, *H*_fin_, length (*L*_G_) of gates (G1 ∼ G4), and *p*-type doping concentration in the floating-fin body except for the anode and cathode are 10, 40, and 14 nm and 2 × 10^15^ cm^-3^, respectively. The gate stack formed between the gate and channel consists of SiO_2_ (2 nm)/Si_3_N_4_ (4 nm)/SiO_2_ (6 nm). When a *V*_G2_ of <-3 V, a *V*_G3_ of >3 V, and a *V*_G4_ of >3 V, *I*_A_ show rapid switching as *V*_G1_ increases. Note that the hole and electron injection barriers (*n*-type and *p*-type) are formed by the appropriate biases (*V*_G2_ ∼ *V*_G4_).

### I&F Function of the PF Device

**FIGURE 7:**
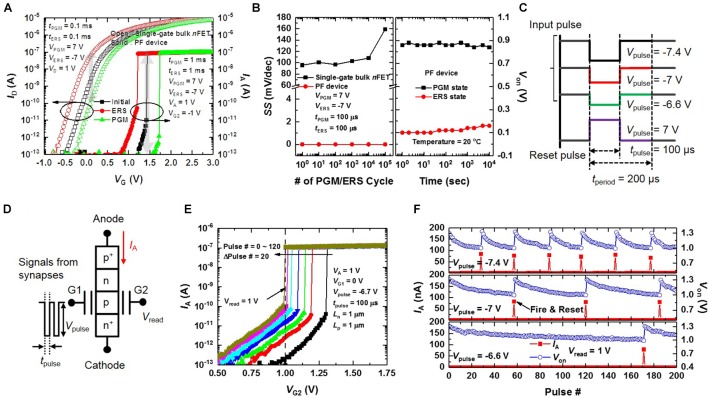
Integrate-and-fire (I&F) function of the fabricated PF device with a charge trapping capability. **(A)** *I*_D_–*V*_G_ and *I*_A_–*V*_G1_ curves of the fabricated single-gate bulk *n*FET and PF device in the initial, program (PGM) and erase (ERS) states. When the shift in *V*_on_ (*V*_th_) of the PF device (the single-gate bulk *n*FET) between the PGM and ERS states is 0.5 V, the *I*_on_/*I*_off_ ratio of the PF device is much larger than that of the single-gate bulk *n*FET. **(B)** *SS* change of the fabricated single-gated bulk *n*FET and PF device with PGM/ERS cycling (left), and retention characteristics of the fabricated PF device at room temperature (right). **(C)** Waveforms of the input pulses with different amplitudes and a reset pulse. **(D)** Schematic of the PF device for I&F operation. **(E)** *I*_A_–*V*_G2_ curves of the fabricated PF device as a parameter of the pulse #. **(F)** Repeatability of *V*_on_ and *I*_A_ with the pulse # as a parameter of *V*_pulse_. When a large |*V*_pulse_| is applied to G1, the integration rate is fast, and the fire rate also increases. All measurements were performed on the same device.

**Figure 7A** compares the *I*–*V* curves of a fabricated single-gate bulk *n*FET and the PF device with the charge trap layer in the initial, program (PGM) and erase (ERS) states. Here, the *W* and *L* of the single-gate bulk *n*FET are 35 nm and 1 μm, respectively. Both *L*_n_ and *L*_p_ of the PF device are 1 μm. When the shift in *V*_on_ (*V*_th_) of the PF device (the single-gate bulk *n*FET) between the PGM and ERS states is 0.5 V, the PF device has a much larger *I*_on_/*I*_off_ ratio than that of the single-gate bulk *n*FET because of the super-steep *SS* property. **Figure 7B** shows the *SS* change of the fabricated single-gate bulk *n*FET and PF device as the PGM/ERS cycling increases from 1 to 10^5^. The *SS* of the PF device does not change while the *SS* of the single-gate bulk *n*FET increases from 95 to 160 mV/dec. Moreover, reasonable retention characteristics of the PF device at the PGM/ERS states are shown in **Figure 7B**. The charge trap function of the proposed PF device (12F^2^) improves the integration density by replacing the integrate-function of the membrane capacitor (*C*_mem_) which occupies a large area in conventional neuron circuits (Chicca et al., 2014). A *C*_mem_ of 0.5 pF has a footprint of 100 μm^2^ (816F^2^) for a 0.35 μm CMOS process (Livi and Indiveri, 2009).

**Figure 7E** demonstrates the I&F function of our PF device by showing the *I*_A_ versus *V*_G2_ curves as a parameter of the number of pulses at a fixed *V*_A_ of 1 V. A pulse bias (*V*_pulse_) of -6.7 V is applied to G1 at the read bias (*V*_read_) applied to G2 of 1 V shown in **Figure 7D**. As the *V*_pulse_ is repeatedly applied to G1, trapped electrons in the charge trap layer are detrapped into the *p*-type Si (electron injection barrier). Then, *V*_on_ of the PF device decreases gradually. When the number of *V*_pulse_ exceeds 120, *V*_on_ becomes less than 1 V leading the device to switch quickly from the off-state to the on-state. Thus, the I&F function is verified in our PF device. **Figure 7F** shows the repeatability of *V*_on_ and *I*_A_ with the number of pulses as a parameter of *V*_pulse_ (see **Figure 7C**) at a *V*_read_ of 1 V. When *V*_on_ is less than 1 V, the PF device fires. After firing, we reset *V*_on_ of the PF device by applying the reset pulse (see **Figure 7C**) to G1 to store electrons in the charge trap layer. As |*V*_pulse_| increases, the fire rate increases because more electrons are detrapped from the charge trap layer into the *p*-type Si at a larger |*V*_pulse_| .

### I&F Neuron Circuit Based on the PF Device

**FIGURE 8:**
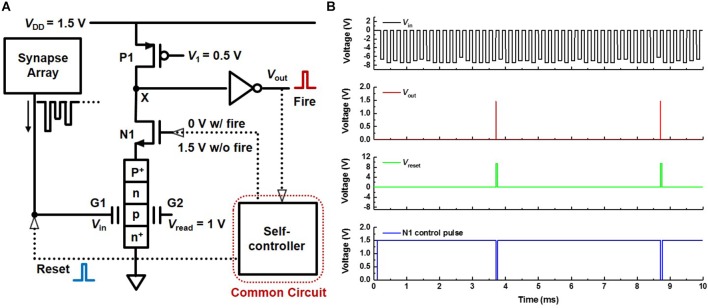
I&F neuron circuit based on the PF device. **(A)** Schematic diagram of the neuron circuit (PF neuron) using the proposed PF device. **(B)** Simulated transient *V*_in_, *V*_out_, *V*_reset_, and N1 control pulse behaviors. Here, *V*_read_ and *V*_1_ are 1 and 0.5 V, respectively.

**Figure 8A** shows a schematic diagram of a neuron circuit (PF neuron) which includes the PF device. Here, the self-controller applies 0 V to the gate of the *n*-MOSFET (N1) when the neuron fires and 1.5 V otherwise. It also applies a reset pulse to the G1 of the PF device when the neuron fires. The operation scheme of the PF neuron is as follows. When signals are repeatedly transmitted from the synapses to the G1 of the PF device, they are integrated into the charge trap layer of the PF device, and the *V*_on_ of the PF device is gradually reduced. At the very moment *V*_on_ becomes lower than *V*_read_, the PF device rapidly switches from the turn-off state to the turn-on state. Then, the voltage at node X changes from a high level to a low level leading to the neuron firing through the inverter. After the neuron fires, N1 is turned off, and the reset pulse is applied to the PF device by the self-controller to return the PF device to its initial state. The simulated transient *V*_in_, *V*_out_, *V*_reset_, and N1 control pulse behaviors of the neuron circuit are shown in **Figure 8B**. These operations verify that the PF neuron mimics effectively the I&F function of the biological neuron.

**FIGURE 9:**
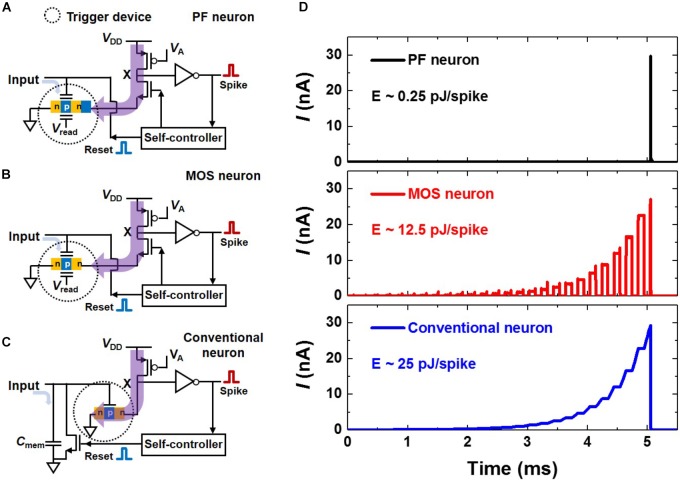
**(A–C)** Schematic diagrams of an I&F circuit (PF neuron) using the PF device with the charge trap layer **(A)**, an I&F circuit (MOS neuron) using a conventional MOSFET with the charge trap layer **(B)**, and a conventional I&F circuit (conventional neuron) with the membrane capacitor (*C*_mem_) **(C)**. **(D)** Transient currents, which flow through the trigger devices in three different neurons, before firing when the same number of input pulses are given to three different neuron circuits.

We perform a simulation to compare the energy consumption of three different neuron circuits shown in **Figures 9A–C**. They are the PF neuron, an I&F circuit using a conventional MOSFET with a charge trap layer (MOS neuron), and a conventional I&F circuit with a *C*_mem_ (conventional neuron). In the PF neuron (or MOS neuron), as the signal from the synapses is transferred to the charge trap layer, the *V*_on,PF_ (or *V*_on,MOS_) decreases gradually. A spike is generated when *V*_on,PF_ (or *V*_on,MOS_) is lower than *V*_read_, which means that the neuron fires. On the other hand, in the conventional neuron, the signal from the synapses is integrated into the *C*_mem_, resulting in a change in the membrane potential (*V*_mem_). If *V*_mem_ exceeds a certain threshold (*V*_th_) in conventional neuron, a spike is generated. **Figure 9D** shows the transient currents before firing when the same number of input pulses are given to the three different neuron circuits. Here, for precise comparisons, *V*_on,PF_, *V*_on,MOS_, or *V*_mem_ changes by 8 mV for each pulse from the synapses, and all neurons are set to generate a spike when 25 pulses are applied. The energies consumed per spike in the conventional neuron and MOS neuron are 25 pJ/spike and 12.5 pJ/spike, respectively. In contrast, the energy consumption per spike in the PF neuron is only 0.25 pJ/spike due to the super-steep *SS* (0.04 mV/dec) of the proposed PF device. For reference, the *SS* of the devices that trigger spike generation in the conventional neuron and MOS neuron is above 60 mV/dec at room temperature. The dotted circle in each neuron represents the trigger device. The trigger device in the PF neuron is the PF device. Note that in the conventional neuron, if *V*_mem_ slowly increases to *V*_th_ or stays at a voltage slightly lower than *V*_th_, the energy consumption is greatly increased because the subthreshold current flows through the trigger device. It should be noted that the same situation also occurs in the MOS neuron. In the proposed PF device, however, the subthreshold current at a lower voltage than the *V*_on,PF_ is negligibly small, which greatly reduces the energy consumption. Moreover, the PF neuron has a foot print of about 52F^2^ except for the self-controller, thereby reducing the neuron area by about 17 times compared with that of the conventional neuron (∼866F^2^) with a large *C*_mem_.

### Multiple Neuron System

**FIGURE 10:**
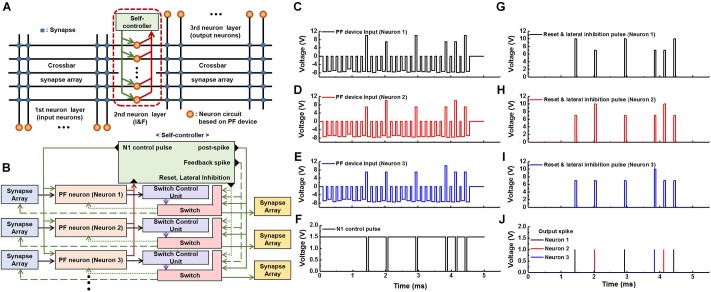
Schematic diagrams and transient behaviors of a multiple neuron system. **(A)** A crossbar architecture with a self-controller in a neuron layer. **(B)** A schematic diagram of a multiple neuron system. **(C–E)** An example of a series of pulses from synapses. This pulse train is applied to a PF device of each PF neuron. Here, positive pulses are reset pulses or lateral inhibition pulses generated from a self-controller. **(F)** The N1 control pulse generated by the self-controller whenever a neuron fires. **(G–J)** Reset and lateral pulses **(G–I)** applied to each neuron whenever a neuron fires **(J)**.

**Figure 10A** shows a crossbar architecture with a common self-controller in a neuron layer. Here, PF neurons and synaptic devices are depicted by the dots and squares, respectively. As in the conventional model of the crossbar layout, input and output neurons are connected by the synaptic devices in a feed-forward manner. Synaptic devices receive a spike signal from the input neurons, and the output neuron receives the signal corresponding to the weighted sum of the synaptic devices. Many neurons in a neuron layer share one common self-controller as depicted by the green box at the top center of **Figure 10A**. **Figure 10B** shows a detailed schematic diagram of a multiple neuron system in a neuron layer depicted by a dashed line in **Figure 10A**. When a spike signal from a fired PF neuron is transmitted into the common self-controller, it generates reset, lateral inhibition, and N1 control pulses. By the switch control unit, reset and lateral inhibition pulses are sent to the fired neuron and remaining neurons except for the fired neuron in the same neuron layer, respectively. The reset pulse returns the PF device in the fired neuron to its initial state, and the lateral inhibition pulse reduces the possibility of firing the remaining neurons to some extent. Note that a positive inhibition pulse is used in our circuit, and its magnitude is designed to be smaller than that of the reset pulse (see **Figures 10G–I**). Additionally, the N1 control pulse is sent to the N1 devices of all the PF neurons, temporarily turning off the N1 devices (**Figure 8A**). Note that by using only one common self-controller in a neuron layer to perform the various functions, the number of transistors in each PF neuron decreases, resulting in high integration density of the neurons.

Transient multiple neurons behaviors in a neuron layer were verified by a circuit simulator as follows. Whenever a PF neuron fires (**Figure 10J**), an N1 control pulse is applied to all neurons to turn off the N1 devices shown in **Figure 10F**. Then, the reset pulse with an amplitude of 10 V is applied to the fired PF neuron, and the lateral inhibition pulse with an amplitude of 7 V is applied to the PF neurons except for the fired neuron shown in **Figures 10G–I**. **Figures 10C–E** show examples of the three pulse sequences from the synapses, respectively. Each pulse train is applied to the PF device of each neuron. Here, positive pulses in each voltage pulse train represent reset (10 V) or inhibition (7 V) pulses transmitted from the self-controller whenever a neuron fires.

### Demonstration of the PF Neuron in a Neural Network

**FIGURE 11:**
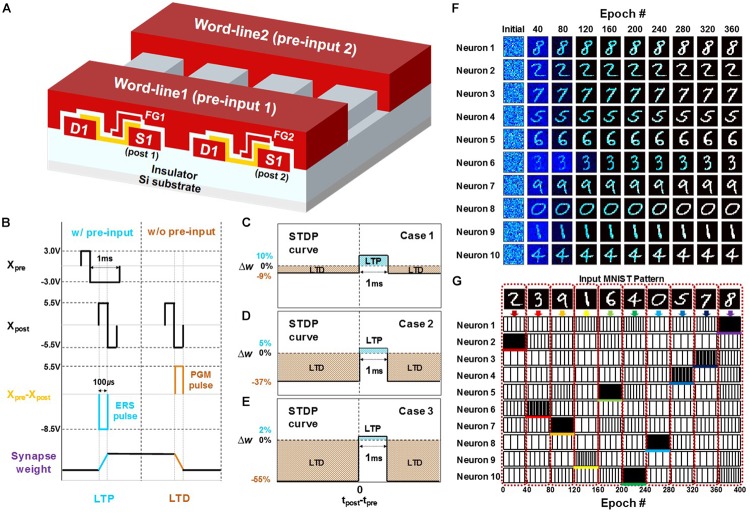
Schematic view of a synapse array and simulation results of the pattern classification based on the spike-timing-dependent plasticity (STDP) rule. **(A)** Three-dimensional schematic view of an array of TFT-type NOR flash memory synaptic devices. **(B)** Pulse scheme from PRE and POST neurons to update the weight of the synapses in a synapse array. **(C–E)** STDP behavior depending on the current weight in a synaptic device when the current weight is low (case 1) **(C)**, moderate (case 2) **(D)**, and high (case 3) **(E)**. **(F)** The evolution of synaptic weights during the learning process. **(G)** Pattern recognition result of neurons after the learning process.

To verify the feasibility of the PF neuron at the network level, on-line unsupervised pattern learning and recognition were investigated in a SNN based on the PF neuron. A fully connected 2-layer SNN system consisting of an input layer of 784 presynaptic neurons and an output layer of 10 postsynaptic neurons was constructed. The pattern classification was performed using a MATLAB simulator. For the simulation, the characteristics of a TFT-type NOR flash memory synaptic device in our previous work (Kim et al., 2018) were adopted for its high performance in a neural network. **Figure 11A** shows a three-dimensional schematic view of an array of TFT-type NOR flash memory synaptic devices. Input signals from presynaptic neurons and a feedback spike pulse from the self-controller in the postsynaptic neuron layer are applied to the word-lines (WLs) and the source, respectively, to perform a long-term potentiation (LTP) and a long-term depression (LTD) by the erasing and programming of the memory cell. The pulse scheme used for the synaptic weight update is shown in **Figure 11B**. When a postsynaptic neuron fires, the weights of the synapse cells to which the input signals are applied are potentiated, and the weights of the other cells without input signals are depressed. The STDP curves derived from the measured LTP/LTD characteristics of the synaptic device by using the pulse scheme are shown in **Figures 11C–E**. **Figure 11C** shows the shape of the STDP curve when the synaptic weight is low. **Figures 11D,E** show the shapes of the STDP curve when the weight of the synapse is moderate and high, respectively.

Pattern classification simulation using the synapse array and the PF neurons is explained as follows. Here, ten binary 28 × 28 input patterns using the MNIST handwritten patterns of the digits 0–9 are used for both learning and recognition. As the input signals of ten MNIST digits are randomly applied to the 784 presynaptic neurons, a postsynaptic neuron of 10 postsynaptic neurons fires by the weighted sum of the synapses, and the weights of synapses in the synapse array connected to the fired postsynaptic neuron are updated by the STDP learning rule. To prevent the co-specialization of postsynaptic neurons, a lateral inhibition function is used by applying the lateral inhibition pulse to all postsynaptic neurons except for the fired postsynaptic neuron. As the unlabeled binary 28 × 28 input patterns are repeatedly presented to the synapse array, each postsynaptic neuron is gradually trained to a specific pattern shown in **Figure 11F**. After the learning process, a recognition test is carried out by randomly presenting the input patterns used in the learning process. Each postsynaptic neuron fires differently in response to each specific pattern shown in **Figure 11G**. The input patterns are recognized by comparing the firing rate of the postsynaptic neurons between 10 postsynaptic neurons resulting in the successful classification of ten MNIST pattern images in an unsupervised learning manner. These simulation results show that the neural network based on our PF neuron successfully performs on-line unsupervised pattern learning and recognition.

## Conclusion

We have demonstrated the I&F function of biological neurons using the proposed body-floated PF device, fabricated on the same wafer with body-connected CMOS FETs for high a performance neuron circuit. The *SS* obtained from the fabricated PF device was excellent at 0.04 mV/dec due to the PF in the switching, resulting in a low energy consumption of ∼0.25 pJ per spike of the neuron circuit using the PF device. The energy consumption is about 100 times less than that of the conventional neuron circuit. The PF neuron area was reduced by about 17 times compared to that of the conventional neuron by replacing a large *C*_mem_ in the conventional neuron to the charge trap layer of the PF device keeping the integrate function of biological neurons. Furthermore, the high density multiple neuron system with reset and lateral inhibition functions was demonstrated through simulation by using only one common self-controller in a neuron layer. We have successfully verified the on-line unsupervised pattern learning and recognition in the SNN system using our PF devices and the synaptic devices. These results show the feasibility of a hardware-based implementation of energy-efficient and high-density analog computing using our PF device.

## Author Contributions

KB, SW, and JH conceived and designed the experiments. KB and SW built the devices and carried out the measurements. KB, SW, and WM performed the circuit simulation. SOL, CH, and SUL performed the pattern learning and recognition simulation. KB, SW, WM, SOL, CH, JH, SUL, and JH performed the theoretical analyses. KB, SW, WM, SOL, CH, and JH wrote the manuscript. All authors discussed the results and commented on the manuscript.

## Conflict of Interest Statement

The authors declare that the research was conducted in the absence of any commercial or financial relationships that could be construed as a potential conflict of interest. The reviewer NM and handling Editor declared their shared affiliation.
