# Supplementary figures and images for: A Split-Gate Positive Feedback Device With an Integrate-and-Fire Capability for a High-Density Low-Power Neuron Circuit
Source: Front Neurosci. 2018 Oct 9;12:704. doi: 10.3389/fnins.2018.00704 (PMC6189404; doi:10.3389/fnins.2018.00704)

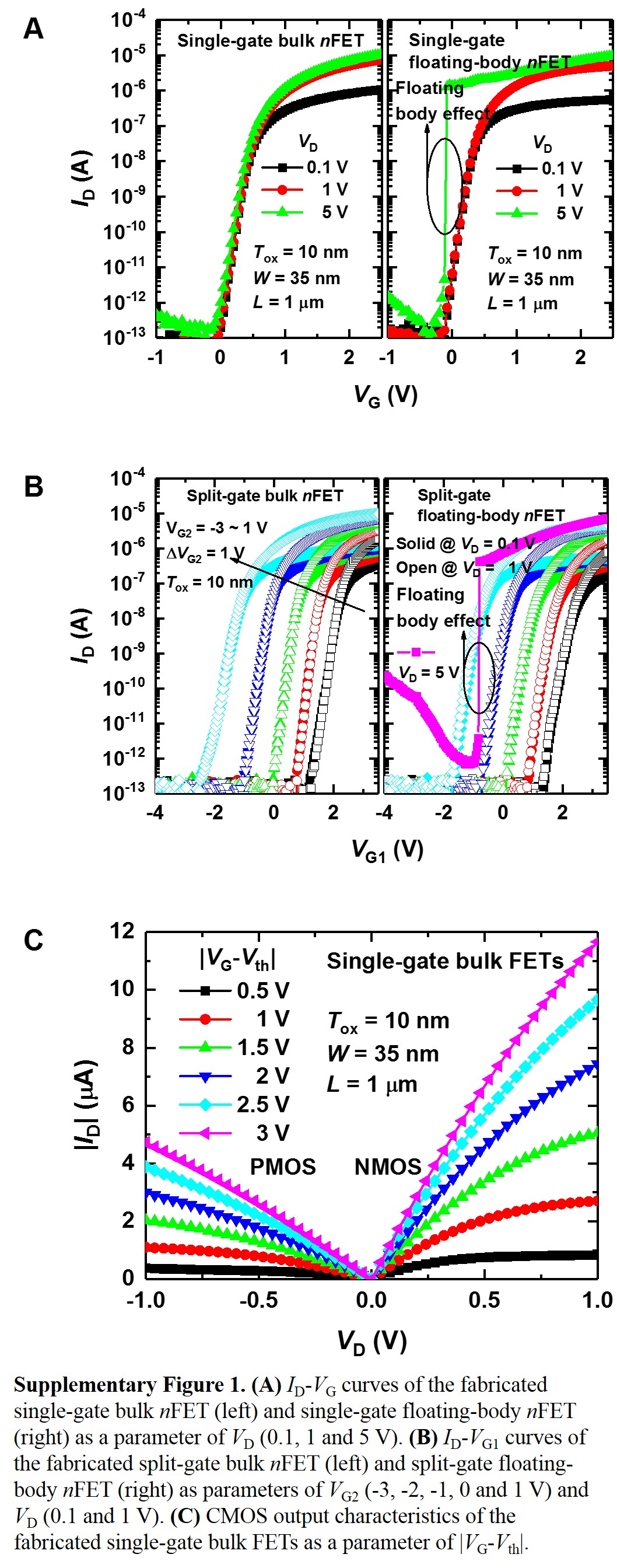

Supplement: Supplementary file 1 [file Image_1.JPEG]
